# Diagnostic value of apparent diffusion coefficient in predicting pathological T stage in patients with thymic epithelial tumor

**DOI:** 10.1186/s40644-022-00495-x

**Published:** 2022-10-05

**Authors:** Chao-Chun Chang, Chia-Ying Lin, Li-Ting Huang, Ming-Tsung Chuang, Ying-Hung Lu, Wei-Li Huang, Ying-Yuan Chen, Wu-Wei Lai, Yau-Lin Tseng, Yi-Ting Yen

**Affiliations:** 1 Division of Thoracic Surgery, Department of Surgery, National Cheng Kung University Hospital, College of Medical College, National Cheng Kung University, Tainan, Taiwan; 2 Department of Medical Imaging, National Cheng Kung University Hospital, College of Medical College, National Cheng Kung University, Tainan, Taiwan; 3 Division of Trauma and Acute Care Surgery, Department of Surgery, National Cheng Kung University Hospital, College of Medical College, National Cheng Kung University, Tainan, Taiwan

**Keywords:** Apparent diffusion coefficient, Diffusion-weighted imaging, Pathological T staging, Thymic epithelial tumor

## Abstract

**Purposes:**

This study aimed to evaluate the diagnostic capacity of apparent diffusion coefficient (ADC) in predicting pathological Masaoka and T stages in patients with thymic epithelial tumors (TETs).

**Methods:**

Medical records of 62 patients who were diagnosed with TET and underwent diffusion-weighted imaging (DWI) prior to surgery between August 2017 and July 2021 were retrospectively analyzed. ADC values were calculated from DWI images using *b* values of 0, 400, and 800 s/mm^2^. Pathological stages were determined by histological examination of surgical specimens. Cut-off points of ADC values were calculated via receiver operating characteristic (ROC) analysis.

**Results:**

Patients had a mean age of 56.3 years. Mean ADC values were negatively correlated with pathological Masaoka and T stages. Higher values of the area under the ROC curve suggested that mean ADC values more accurately predicated pathological T stages than pathological Masaoka stages. The optimal cut-off points of mean ADC were 1.62, 1.31, and 1.48 × 10^–3^ mm^2^/sec for distinguishing pathological T2-T4 from pathological T1, pathological T4 from pathological T1-T3, and pathological T3-T4 from pathological T2, respectively.

**Conclusion:**

ADC seems to more precisely predict pathological T stages, compared to pathological Masaoka stage. The cut-off values of ADC identified may be used to preoperatively predict pathological T stages of TETs.

**Supplementary Information:**

The online version contains supplementary material available at 10.1186/s40644-022-00495-x.

## Introduction

Thymic epithelial tumors (TETs) belong to a heterogeneous family of anterior mediastinal tumors [1]. TETs are the most common tumor arising from the thymus with an incidence of 1.3 to 3.2 per million worldwide [2]. The most predominant types of TETs are thymoma and thymic carcinoma [3]. Compared with thymoma, thymic carcinoma is more aggressive with a worse prognosis [1, 4]. Patients with thymic carcinoma often undergo surgery plus adjuvant radiotherapy and/or chemotherapy [2, 5]. Based on the World Health Organization (WHO) histological classification, thymomas are further classified into low-risk thymomas (A, AB, and B1) and high-risk thymomas (B2 and B3) [2, 6]. Most patients with low-risk thymoma receive surgery alone, whereas patients with high-risk thymomas might require adjuvant treatments [5, 7].

Preoperative differential diagnosis and staging of TETs are clinically essential for facilitating the therapeutic decision-making process, thereby improving prognosis [2, 8]. TETs are classified based on their histopathologic characteristics and staged by the presence and extent of invasion, implants, lymph node involvement, and distant metastases [9]. Classification by staging is important for TET management. The Masaoka staging system and the tumor nodes metastasis (TNM) staging system are used extensively for clinical and/or pathological staging of TETs [6, 8, 10, 11], which guide therapy, evaluate the results of surgery, and provide patient prognosis. The further breakdown of stages to T, N, and M categories allows detailed reporting of TET beyond what is available in the Masaoka-Koga system [12].

Several lines of evidence have examined the potential of apparent diffusion coefficient (ADC) values derived from diffusion-weighted imaging (DWI) in predicting pathological subtypes and stages (TNM and Mosaoka) of TETs [6, 10, 13–15]. Briefly, conventional MRI can only speculate on the degree of tumor tissue invasion based on morphology, and it may not be able to accurately distinguish low risk and high risk TETs [13, 16]. The ADC value indicates the rate of water diffusion within tissue [10], and provides an objective basis for judgment, avoiding the influence of individual morphological differences on subjective interpretation. A study of 57 patients with TETs observed that mean ADC values were 1.63 × 10^–3^, 1.30 × 10^–3^, and 0.86 × 10^–3^ mm^2^/s for low-risk thymoma, high-risk thymoma, and thymic carcinoma, respectively [15]. ADC cutoff values were used to discriminate low-risk thymoma from high-risk thymoma [6, 13, 14], and thymic carcinoma [13, 14]. Another study of 37 TET patients demonstrated the potential of ADC in differentiating early Masaoka stage of TETs (stage I and II) from advanced Masaoka stage (stage III and IV) [14]; however, another study of 41 thymoma patients reported inconclusive findings on predicting Masaoka classification of thymomas [6]. In contrast, the correlation between ADC and TNM staging of TETs remains to be explored.

Given the increasing role of ADC values in the differential diagnosis of TETs [10, 13–15], the purpose of this retrospective study was to evaluate the differential diagnostic value of ADC in predicting pathological T stages (TNM or Masaoka staging system) in patients with TETs.

## Methods

### Patient population

This retrospective study was approved by the Institutional Review Board of National Cheng Kung University Hospital, and informed consents from participants were waived due to the retrospective nature of the study. Consecutive patients who were diagnosed with TET more than 2 cm at the longest diameter from August 2017 to July 2021 were initially selected. Inclusion criteria were (i) undergoing DWI prior to radical surgery to remove TET; (ii) histological examination of surgical specimens for complete pathologic staging; and (iii) patient did not undergo biopsy, neoadjuvant chemotherapy, or radiation therapy prior to MRI exam. Patients with unresectable TET were excluded.

### Study variables

Study variables were collected from medical records, including age, sex, the presence of myasthenia gravis, and the results of clinical and pathological staging. According to the TNM staging system, clinical T stages were determined based on the morphology of TET derived from T2WI, non-contrast, and contrast-enhanced T1WI imaging as previously described [17]. Three classification systems of TET were used to determine pathological stages, including the 2015 WHO classification, the 1994 Masaoka-Koga staging system, and the 8^th^ edition of the TNM staging system as previously described [9]. ADC values were calculated from digital MRI images obtained with varying degrees of diffusion weighting (*b* values = 0, 400, 800 s/mm^2^) [13].

### Mediastinal MRI protocol

All patients underwent chest MRI examination using a 3 Tesla MRI system (Ingenia, Philips Healthcare, Best, Netherlands) with axial multi-echo Dixon (mDixon, and in-phase, out-of-phase, fat-only, and water-only imaging), electrocardiogram-gated double inversion recovery T2-weighted sequence, and pre- and post-contrast enhanced fat-suppressed T1-weighted imaging.

Diffusion-weighted images were obtained by using multi-section, single-shot, breath-hold spine-echo, echo-planar imaging sequences (TR/TE = 2735/79 ms; slice thickness = 7 mm; interslice gap = 1 mm; voxel size = 3 mm × 3.02 mm; field of view (FOV) = 400 mm × 350 mm). DWI was acquired with *b* values of 0, 400, and 800 s/mm^2^ and ADC maps were generated automatically on the MRI console. Protocol details are shown in Supplementary Table S1.

### Imaging analysis

Tumor boundaries were delineated via a combination of T1-weighted, T2-weighted, contrast enhanced T1-weighted, and DWI. Two readers (a radiologist and a thoracic surgeon with 7 years of experience in chest imaging), who were blinded to patient’s medical records, independently delineated regions of interest (ROIs) using a commercial workstation with standard software (IntelliSpace portal, version 6). A freehand ROI was placed on the ADC map on three consecutive slices where the largest area of the tumor was included. If there was a difference in tumor location on the pre-, post-contrast imaging, and ADC map after imaging registration, it was manually assessed by the radiologist or thoracic surgeon based on the ADC map. ROI size was kept as large as possible, avoiding necrotic parts, blood vessels, or interference from the surrounding tissue. The ADC values obtained from 3 ROIs were averaged and then used for the final statistical analysis.

### Statistical analysis

Patients’ demographic and clinical characteristics are expressed as mean ± standard deviation for age, and as n and percentage (%) for other variables. The distribution of mean ADC values is presented as median and interquartile range. The Kruskal–Wallis test was used to determine the statistical significance of intergroup differences in various pathological stages with ADC. Receiver operating characteristic (ROC) analysis was performed, and the area under the curve (AUC) was used to evaluate the predictive ability of ADC values for pathological Masaoka stages and pathological T stages. Differences in AUC between pathological Masaoka stages and pathological T stages were examined using DeLong’s test. According to ROC curves, cut-off points of ADC values with corresponding predicted sensitivity and specificity for distinguishing pathological T stages were calculated based on the statistics of true positive rate + (1-false positive rate).

Inter-observer reliability was evaluated with the intraclass correlation coefficient in a two-way random-effects model of absolute agreement [18]. All statistical assessments were two-tailed and considered significant at *p* < 0.05. Statistical analyses were performed using IBM SPSS statistical software version 22 for Windows (IBM Corp., Armonk, NY, USA).

## Results

### Study population

In this retrospective study, 74 patients with TETs were initially selected. Of them, 12 patients who had unresectable TET were excluded. As a result, 62 TET patients who underwent DWI examination prior to radical surgery were subjected to the final statistical analysis.

The demographic and clinical characteristics of eligible patients are summarized in Table 1. The study population had a mean age of 56.3 years and consisted of 33 males and 29 females. Eleven out of 62 eligible patients had myasthenia gravis. The mean pre-operative tumor size was 6.11 cm. Based on clinical T staging, 26, 12, 22, and 2 patients had clinical T1, T2, T3, and T4, respectively. Pathological examination revealed that 48 patients had thymoma and 14 patients had thymic carcinoma, and that the most common WHO histologic subtype was B2 (32.3%), followed by AB (22.6%), thymic carcinoma (22.6%), A (12.9%), B1 (6.5%) and B3 (3.2%). According to the Masaoka staging system, 21, 17, 11 and 13 patients had pathological Masaoka stage I, II, III, and IV, respectively. Based on the TNM staging system, the numbers of patients diagnosed with pathological T1, T2, T3, and T4 stages were 38, 4, 13, and 7, respectively. Furthermore, 11 patients were diagnosed with pathological M1a stage and 2 patients had M1b stage. Regarding N staging, there was only one patient diagnosed with pathological N2 stage (Table 1).

**Table 1:** Demographic and clinical characteristics of 62 patients with thymic epithelial tumor

Variables	(*N* = 62)
Sex, males	33 (53.2%)
Age, years	56.31 ± 12.80
Myasthenia gravis	11 (17.7%)
Pre-operative tumor size, cm	6.11 ± 3.83
Clinical T staging	
T1	26 (41.9%)
T2	12 (19.4%)
T3	22 (35.5%)
T4	2 (3.2%)
Pathological WHO histological classification	
A	8 (12.9%)
AB	14 (22.6%)
B1	4 (6.5%)
B2	20 (32.3%)
B3	2 (3.2%)
Carcinoma	14 (22.6%)
Pathological Masaoka staging	
I	21 (33.9%)
II	17 (27.4%)
III	11 (17.7%)
IV	13 (21%)
Pathological T staging	
T1	38 (61.3%)
T2	4 (6.5%)
T3	13 (21%)
T4	7 (11.3%)
Pathological M staging	
M1a	11 (17.7%)
M1b	2 (3.2%)

N stage was not shown because only one patient was N2

### Apparent diffusion coefficient values

After all eligible patients were stratified by pathological Masaoka stage, mean ADC values were significantly different among 4 pathological Masaoka stages (*p* < 0.001; Table 2). Similarly, after re-stratifying all eligible patients by pathological T stage, mean ADC values were also significantly different among the 4 pathological T stages (*p* < 0.001; Table 2).

**Table 2:** Distribution of ADC values for pathological Masaoka stage and pathological T stage

	ADC value	*P* value
Pathological Masaoka stage		< .001*
I	2.09 (1.55, 2.66)	
II	1.91 (1.53, 2.31)	
III	1.32 (0.97, 1.98)	
IV	1.03 (0.82, 1.51)	
Pathological T stage		< .001*
T1	1.99 (1.59, 2.47)	
T2	2.10 (1.65, 2.59)	
T3	1.16 (0.91, 1.44)	
T4	0.97 (0.79, 1.23)	

ADC value is expressed in 10^–3^ mm^2^/sec

Data are presented as median and interquartile range, and differences among pathological stages were examined using Kruskall-Wallis test

^*^P < .05, significantly different among pathological stages

Furthermore, the AUC values for distinguishing pathological Masaoka stages or pathological T stages were determined via ROC analyses (Table 3). The AUC values for differentiating pathological T stages were greater than those for distinguishing pathological Masaoka stages, however, no significant differences in AUC between groups were observed. These findings suggested that mean ADC values might be more accurate in predicting pathological T stages (Table 3).

**Table 3:** The AUC values for discriminating pathological Masaoka stages or pathological T stages

Pathological Masaoka stages	AUC	Pathological T stages	AUC	*P* value
M2-M4 vs. M1	0.736	T2-T4 vs. T1	0.821	0.34
M4 vs. M1-M3	0.835	T4 vs. T1-T3	0.908	0.37
M3-M4 vs M1-M2	0.821	T3-T4 vs T1-T2	0.896	0.32

*AUC* Area under the curve

**Fig. 1:**
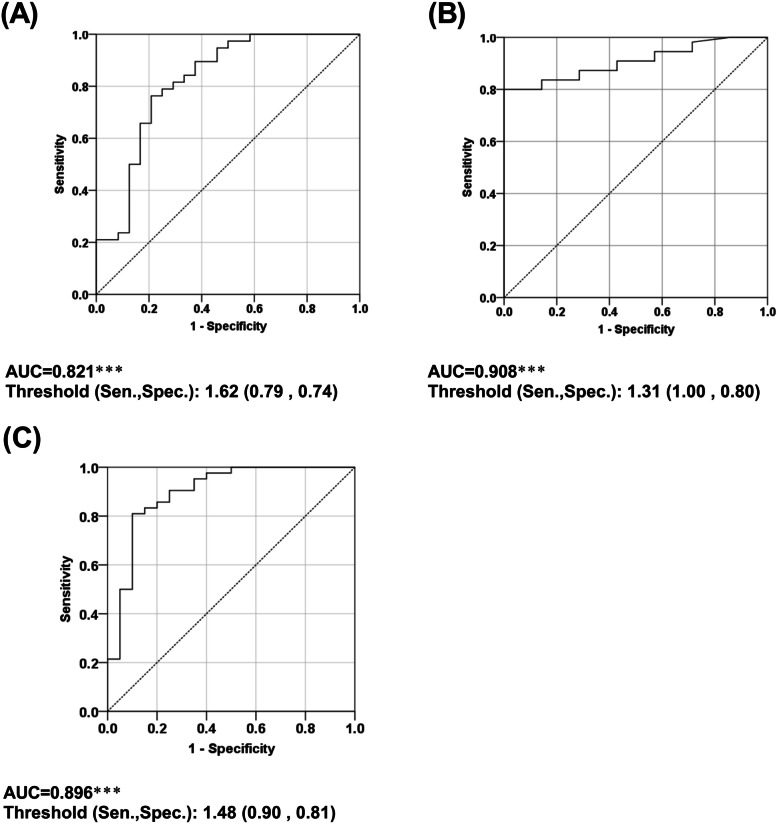
ROC curves of ADC for distinguishing (**A**) pathological T2-T4 vs. pathological T1 (**B**) pathological T4 vs. pathological T1-T3 (**C**) pathological T3-T4 vs. pathological T1-T2 Results were summarized as AUC and threshold (derived Sen., Spec.). Abbreviations: ROC, received-operative characteristic; AUC, area under ROC curve; Sen., Sensitivity; Spec., specificity. ^*^P < .05, ^**^P < .01, ^***^P < .001, indicated significant ROC analysis

The effectiveness of mean ADC values in discriminating pathological T stages of TETs was further explored. The ROC analysis showed that the mean ADC value was significantly associated with distinguishing pathological T2-T4 from pathological T1, pathological T4 from pathological T1-T3, and pathological T3-T4 from pathological T2 (AUC = 0.821, 0.908, and 0.896, respectively; all *p* values < 0.001; Fig. 1A to C). The thresholds, which were calculated based on the derived cut-off points of mean ADC values, were 1.62, 1.31, and 1.48 for distinguishing pathological T2-T4 from pathological T1, pathological T4 from pathological T1-T3, and pathological T3-T4 from pathological T2, respectively (Fig. 1A to C).

**Fig. 2:**
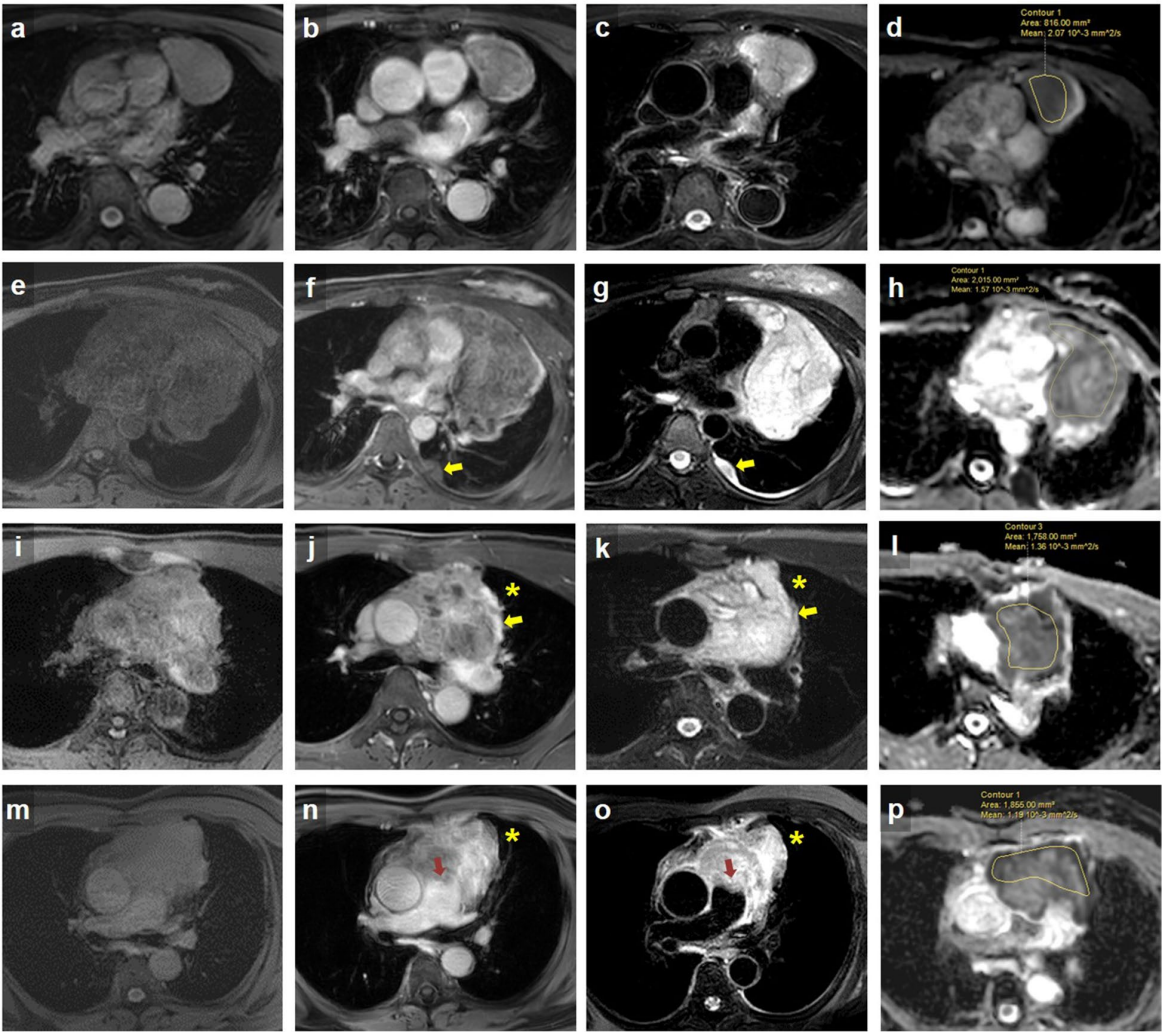
Representative MR images of TETs with various T stages. **a-d** A 65-year-old male with T1 type B2 thymoma. Axial pre-, post-gadolinium T1WI (**a, b**) and black-blood T2WI (**c**) showed a well-defined tumor in the prevascular mediastinum. The freehand region of interest (ROIs) were manually drawn on the ADC map, and the ADC value was 2.07 × 10^–3^ mm^2^/sec (**d**). He underwent video-assisted thoracoscopic surgery/ thymothymectomy. **e–h** A 33-year-old female with T2 type AB thymoma. Axial pre-, post-gadolinium T1WI (**e, f**) and T2WI (**g**) showed a lobulated tumor in the prevascular mediastinum with pericardial invasion and left pleural seeding tumor (arrow). The ADC value was 1.57 × 10^–3^ mm^2^/sec (**h**). She underwent left thoracotomy thymectomy and pleural tumor excision. **i-l** A 41-year-old male with T3 thymic carcinoma. Axial pre-, post-gadolinium T1WI (**i, j**) and T2WI (**k**) showed an irregular tumor with the left upper lobe (LUL) lung (asterisk) and extrapericardial pulmonary artery (PA) invasion (arrow). The ADC value was 1.36 × 10^–3^ mm^2^/sec (**l**). He received neoadjuvant concomitant chemoradiotherapy and underwent full sternotomy thymothymectomy, left mini-thoracotomy with rib spreading LUL lobectomy. **m-p** A -64-year-old male with T4 thymic carcinoma. Axial pre-, post-gadolinium T1WI (**m, n**) and T2WI (**o**) showed an irregular tumor with intrapericardial PA (arrow) and LUL lung invasion (asterisk). The ADC value was 1.19 × 10^–3^ mm^2^/sec (**p**). He received neoadjuvant concomitant chemoradiotherapy, followed by underwent thymothymectomy, partial pericardiectomy, cardiopulmonary bypass for pulmonary trunk reconstruction with Bovine patch, and LUL wedge resection

The optimal ADC cut-off values were used to evaluate the total accuracy for predicting pathological T stage based on mean ADC values. The total accuracy for mean ADC values was 64.5% when cut-off values were set as 1.62, 1.48, and 1.31 (Table 4). In addition, the representative MR images of different T stages of TETs are presented in Fig. 2.

**Table 4:** Accuracy of predicting pathological T stage based on cut-off ADC values

		Clinical T stage					Predicted pathological T stage				Cut off of ADC value
		T1	T2	T3	T4		T1	T2	T3	T4	
Pathological T stage	T1	23	8	6	1		28	2	4	4	> 1.62
	T2	2	0	2	0		3	1	0	0	> 1.48
	T3	1	4	8	0		2	0	4	7	> 1.31
	T4	0	0	6	1		0	0	0	7	
**Accuracy**						**51.6%**					**64.5%**

The predicted results were expressed as number of patients

The total accuracy was defined as the percentage of patients on diagonal cells (shaded cells) among 62 included patients

### Reproducibility assessmen

The intraclass correlation coefficient was 0.917 for the measurement of mean ADC values, suggesting excellent inter-observer reproducibility.

## Discussion

The potential of ADC values derived from DWI in predicting pathological subtypes and stages (TNM and Mosaoka) of TETs has been previously reported [6, 10, 13–15]. Considering the possible role of ADC values in differential diagnosis and predicting pathological subtypes and stages of TETs [10, 13–15], the purpose of this retrospective study was to evaluate the potential of ADC for predicting pathological T stages or pathological Masaoka stage in patients with TETs. The present study demonstrated that pathological T stages were negatively correlated with mean ADC values in TETs. Based on the ADC cut-off values derived from ROC analyses, ADC values seemed to more accurately distinguish pathological T stages of TETs compared with Masaoka stages. Moreover, two distinct sets of optimal cut-off values of mean ADC for differentiation between pathological T stages of TETs were identified.

Dynamic water diffusion within tissues is affected depending on membrane permeability, cellular volume fraction, and tissue microstructure [10, 19], and is quantitatively expressed as ADC at varied *b* values [20]. Since ADC changes reflect the pathophysiological deficit, ADC was used to discriminate malignant from benign mediastinal tumors [21, 22], and to monitor long-term therapeutic responses in mediastinal lymphadenopathy and breast cancer [23, 24]. Notably, the *b* value indicates the strength and timing of the gradients in DWI, which influences the sensitivity to diffusion-based contrast [25] and the subsequent calculation of ADC values [13, 25]. A range of *b* values (0 to 800 s/mm^2^) were commonly used in studies exploring the diagnostic ability of ADC in distinguishing subtypes and stages of TETs [6, 13, 18, 26]. Worth noting, no contrast agent (eg, gadolinium) was used for DWI in the current study, as contrast medium can have a significant impact on ADC value in a time and lesion-type dependent manner, as well as the quality of images [27, 28].

A study using a 1.5 T MR scanner found that mean ADC values were 1.43 ± 0.26 and 1.31 ± 0.31 × 10^–3^ mm^2^/sec for early and advanced Masaoka stages of thymomas, respectively [6]. The present study with a 3 T MR scanner observed that TETs with more advanced pathological Masaoka stages had lower mean ADC values [6]. Another study utilizing a 3 T MR scanner reported that the optimal cut-off value in discriminating between advanced and early Masaoka stages may be the 10^th^ percentile of ADC values [14]. Thus, the variability between the current results with those of others may be in part due to distinct MR scanners, *b* values and/or ADC measurements used [6, 14].

Masaoka stages I and II of TETs are resectable, but Masaoka stage III overlaps with stages T2 to T4, including unresectable and resectable TETs [8]. T2 stage TETs with pericardium invasion may be managed by thoracoscopy. T3 stage TETs often require full sternotomy. T4 stage TETs with intrapericardial pulmonary artery invasion and aortic invasion require cardiopulmonary bypass during surgery and are considered inoperable. In contrast, the degree of invasion to different organs is classified into various T stages in the TNM staging system [8]. Masaoka stage IV reflects only M1 stage of the TNM staging system and cannot differentiate resectable TETs from unresectable TETs. For example, T3N0M1a is equivalent to Masaoka stage IV and it is recommended to actively remove all visible tumors according to the current guideline for TNM staging. TNM stages are significantly correlated with WHO histological classification in TETs, and its prognostic significance is suggested [11]. Thus, compared with pathological Masaoka stages, preoperatively predicting pathological T stage may provide more valuable information to facilitate the selection of optimal therapeutic strategies.

Clinical T staging is an important component of assessing risk and managing patients with TET. Inconsistencies in clinical T staging and pathological T staging can lead to unwanted variability in the selection of surgical methods thus impacting the extent of resection that may be required. Currently, clinical T staging largely depends on preoperative CT or conventional MRI; however, considerable inconsistencies still exist in pathological T staging. The addition of preoperative ADC values as part of clinical T staging may improve consistency, which warrants further investigation. Regarding ADC and WHO pathological staging, a study of thymic epithelial tumors has previously confirmed the correlation between ADC value and WHO pathological classification [13]. However, the WHO pathological classification, compared with the TNM staging system or the Masaoka staging system, is poorly correlated with prognosis and is of little clinical significance [29, 30].

To our knowledge, this study is the first to demonstrate a negative correlation between ADC values and the pathological T stages in patients with TETs. A previously published database study of 907 TET patients revealed that the T stage was a predictive factor for recurrence of TETs [31], so predicting the pathological T stage preoperatively is of clinical significance. In this study, only the pathological T stages were assessed because all four T stages were found in the study population. Given that the distribution of different WHO histological types in different T stages was uneven in the current study, pathological T1 and T3 were assessed to determine whether differences existed with WHO histological classification. A preliminary post-hoc analysis revealed no significant difference in ADC value of different pathological histological types under the same pathological T stage, although the ADC value of the rare pathological T1 stage thymic carcinoma was lower. TETs with the same pathological T stage, but different pathological WHO histological types may have different thresholds; however, in clinical practice, the pathological T1 or T2 thymic carcinoma and the pathological T3 or T4 type A thymoma are very rare. Larger scale multicenter studies are warranted to further address this question considering any limitations associated with output from a single institution study with a small number of patients.

Future experiments should investigate whether the addition of preoperative ADC values for clinical T staging can improve the consistency between clinical T staging and pathological T staging. In addition, optimal cut-off values of mean ADC were identified. The AUC values for discriminating pathological T stages were between 0.821 and 0.908 in this single institution retrospective study, suggesting preoperative ADC has promising diagnostic value in predicting pathological T stages in patients with TET.

The predictive accuracy of diagnostic imaging is critical for differential diagnosis of TETs, and has been improved via multiparametric chest MR, such as the combination of DWI and perfusion-weighted imaging [10], and the radiomics nomogram including conventional MR imaging parameters, ADC value, and radiomic signatures [32]. Moreover, ADC and texture parameters derived from DWI were used together to predict pathological WHO types and Masaoka stages of TETs [3].

Limitations in the current study need to be addressed. First, this research is a single institution study with a small number of patients, so larger-scale multicenter studies are warranted to confirm current findings. Second, T4 invasion usually is unresectable, and only one patient with clinically suspicious T4 TET underwent surgery; therefore, selection bias cannot be excluded. Third, additional imaging parameters will be included in the future to precisely discriminate pathological T3 from T4 TETs. Fourth, small tumors may have been overlooked because it is difficult to identify and measure tumors less than 2 cm due to the potential imaging artifacts. Finally, it should be recognized that the optimal ADC cut-off values may vary, depending on the MRI vendor and software.

## Conclusions

The current study demonstrated the negative correlation between ADC and pathological T stages, and the diagnostic value of ADC to differentiate between pathological T stages of TETs. The current findings may facilitate the development of accurate diagnostic imaging for predicting pathological T stages of TETs, thereby improving the differential diagnosis and prognosis of TETs.

## Supplementary Information

**Additional file 1:** **Supplementary Table 1.** MRI acquisition parameters.

## Data Availability

All data generated or analyzed during this study are included in this published article and its supplementary information files.

## Abbreviations

ADC: Apparent diffusion coefficient
AUC: Area under curve
DWI: Diffusion-weighted imaging
ROC: Receiver-operative characteristic
ROI: Region of Interest
TET: Thymic epithelial tumor
WHO: World Health Organization
