# Supplementary material for: Diagnostic value of apparent diffusion coefficient in predicting pathological T stage in patients with thymic epithelial tumor
Source: Cancer Imaging. 2022 Oct 5;22:56. doi: 10.1186/s40644-022-00495-x (PMC9533584; doi:10.1186/s40644-022-00495-x)
Supplement: Supplementary file 1 — Additional file 1: Supplementary Table 1. MRI acquisition parameters. [file 40644_2022_495_MOESM1_ESM.docx]

**Supplementary Table 1. MRI acquisition parameters**

| **Sequences** | **Scan plane** | **Voxel size (mm)** | **Slice thickness/ gap (mm)** | **TR/TE (ms)** | **FoV (mm)** | **Flip angle (^o^)** |
| --- | --- | --- | --- | --- | --- | --- |
| mDIXON (water, fat, in-phase/ out-of-phase) | Axial | 1.5 x 1.5 | 6/-3 | 3.7/ 1.32/ 2.4 | 300 x 300 | 15 |
| T2W TSE FS | Sagittal | 1.2 x 1.7 | 8/ 1 | 800/ 71 | 350 x 328 | 90 |
| Cardiac-gated Double IR T2W | Axial | 1.5 x 1.5 | 7/ 1 | shortest/ 73 | 250 x 250 | 90 |
| DWI (b= 0, 400, 800 sec/mm^2^) | Axial | 3 x 3.02 | 7/ 1 | 2735/ 79 | 400 x 350 | 90 |
| T1W FS | Axial | 1.5 x 1.5 | 6/-3 | 3.6/ 1.32 | 300 x 300 | 10 |
| 3D T1-fast field echo (DCE) | Axial | 1.5 x 1.5 | 5/ 0 | 4/2 | 350x257 | 5 and 15 |
| CE T1W FS | Axial | 1.5 x 1.5 | 6/-3 | 3.6/ 1.32 | 300 x 300 | 10 |
| CE T1W FS | Sagittal | 1.3 x 1.3 | 6/-3 | 3.7/ 1.32 | 350 x 280 | 10 |

Abbreviations: TSE = turbo spin echo, FS = fat-suppressed, TE = echo time, TR = repetition time, IR = inversion recovery, DWI = diffusion-weighted imaging, , CE = contrast enhanced
